# Kinesin-14: the roots of reversal

**DOI:** 10.1186/1741-7007-8-107

**Published:** 2010-08-16

**Authors:** Robert A Cross

**Affiliations:** 1 Centre for Mechanochemical Cell Biology, Warwick University Medical School, Gibbet Hill, Coventry CV4 7AL, UK

## Abstract

Kinesin-14 motor proteins step towards microtubule minus ends, in the opposite direction to other kinesins. Work on the still-enigmatic kinesin-14 mechanism published in *BMC Structural Biology *shows that the carboxyl terminus of the motor head undergoes a dock-undock cycle, like that of plus-end-directed kinesins.

See research article: http://www.biomedcentral.com/1472-6807/10/19

## Commentary

Kinesins are the railway engines of the cell, hauling molecular cargo over long distances along microtubule tracks. The kinesin-microtubule railway system is central to the self-organization of eukaryotic life and its mechanisms are consequently an important problem in molecular cell biology. Most kinesins step towards microtubule plus ends, but in one subfamily, the kinesins-14 (K-14), the conventional mechanism is reversed so that the motors haul cargo in the opposite direction, towards microtubule minus ends. Discovering the reversal mechanism is proving challenging. A new crystal structure of a kinesin-14 point mutant [1] now visualizes for the first time the docking of the proximal part of the kinesin-14 carboxyl terminus to a site on the main part of the kinesin head, showing that the docking and undocking of a carboxy-terminal peptide is a general feature of the mechanism of force generation in both plus-end-directed and minus-end-directed kinesins.

The initial discovery of Ncd [2,3], the first kinesin-14 to be found, stimulated exciting protein-engineering experiments aimed at finding the structural mechanism for backward stepping. It was quickly established that connecting kinesin-14 heads via their carboxyl termini to the tail of kinesin-1 (kinesin-1 is plus-end-directed) results in a plus-end-directed chimera, whereas connecting kinesin-1 heads via their amino termini to a kinesin-14 tail produces a slow minus-end-directed chimera. So far so good, but subsequent experiments revealed a more subtle and complicated picture. Mutating the head-tail junction in kinesin-14 caused it to revert to plus-end-directed motility [4], whereas a point mutation near the head-tail junction (Ncd N340K) produces a schizophrenic motor that drives plus-end-directed microtubule sliding for a few seconds, and then switches to driving minus-end-directed sliding, and vice versa [5]. Making sense of these data requires three-dimensional (3D) structural information. Enter crystallography.

## Structural studies reveal two stable positions of the coiled-coil tail in kinesin-14

**Figure 1:**
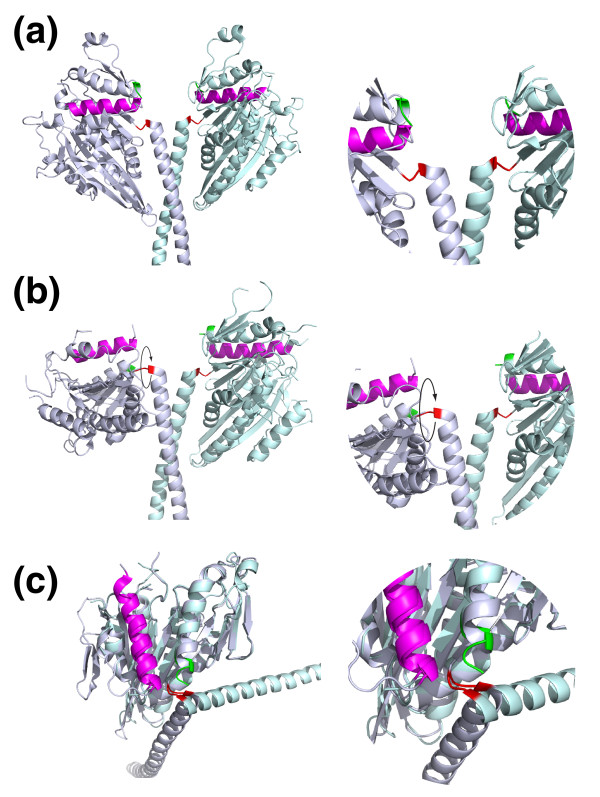
**Kinesin-14 lever positions**. **(a) **A crystal structure of a wild-type Ncd dimer shows a symmetrical structure with the heads in equivalent positions (PDB 1CZ7). The amino terminus of the head is highlighted in red and the carboxyl terminus in green The alpha4 relay helix is magenta. The blow-up on the right of the region linking the motor heads to their tails (the lever) shows the carboxyl termini of the two heads in essentially the same conformation. **(b) **In a dimer formed of mutant Ncd subunits (N600K, PDB 1N6M), one head is rotated relative to the other by about 70°. The new structure of Heuston *et al*. [1] of the mutant T436S shows this arrangement. **(c) **Same data as in (b) above, with one head rotated to overlay the other, showing the coiled coil tail can adopt two stable positions relative to the head.

Crystal structures of wild-type Ncd show the two heads of the dimeric motor in symmetrically equivalent positions (Figure 1a). In the crystal structure of a kinesin-14 mutant (Ncd N600K) [6] this symmetry is broken, with one head in the familiar position, and one rotated by about 70° (Figure 1b). The new structure [1], of a T436S mutant in the active-site P-loop of Ncd (equivalent to T94S in *Drosophila *kinesin-1), shows this same asymmetrical arrangement. Figure 1c displays the same data as for Figure 1b, but with one head of the dimer overlaid on the other, showing that the coiled-coil tail can lie stably in two different positions relative to the motor head. This raises the possibility that flipping between these positions could allow the coiled-coil tail to function as a lever to exert force.

**Figure 2:**
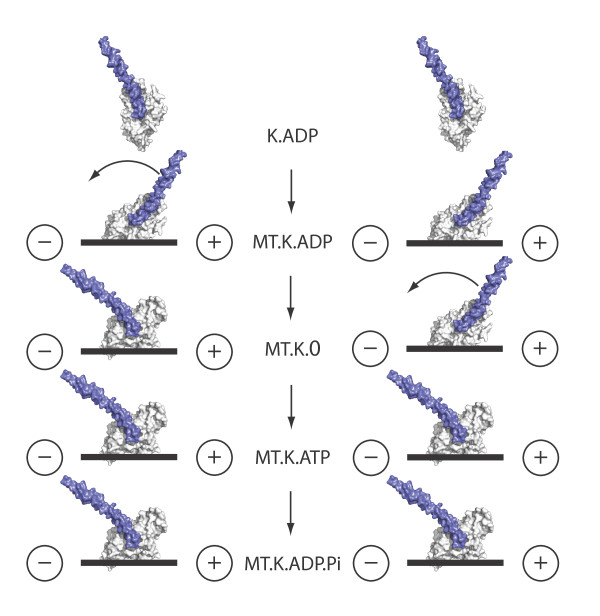
**Competing models for coupling the kinesin-14 lever scheme to ATP turnover**. In one scheme (left), lever motion is coupled to ADP release. In the other (right), it is coupled to ATP binding. K.ADP, kinesin with ADP bound in the active site; MT.K.ADP, kinesin with bound ADP in contact with microtubule (MT); MT.K.0, complex of microtubule and kinesin after ADP release; MT.K.ATP, complex of microtubule and kinesin with bound ATP; MT.K.ADP.Pi, hydrolysis of ATP generates kinesin with ADP and P_i _bound. The - and + signs indicate the minus and plus ends of the microtubule.

Crystallography provides an atomic-resolution view of isolated kinesin heads, but as yet there are no crystal structures of kinesin-tubulin complexes. This is where electron microscopy can help. Kinesins bound to microtubules can now be visualized using 3D cryoelectron microscopy with a resolution sufficient to allow the positions of particular helices and other secondary structural elements to be mapped. Two competing kinesin-14 schemes have emerged, in both of which ATP turnover in the microtubule-attached head drives a lever-swing of the coiled-coil tail. One scheme has this occurring on ATP binding, and the other on ADP release (Figure 2). Lever models are attractive, but also bring new problems: Heuston *et al*. [1] show that the two lever positions are each stabilized by multiple salt bridges and how the lever might be driven from one stable position to another is unclear. Furthermore, the lever (the coiled-coil tail of the motor) is potentially forbiddingly long: perhaps as long as 150 nm in the wild-type molecule, albeit with some regions less stable than others [7].

Confidence in some sort of lever-swing model is nonetheless much increased by experiments showing that the ability of Ncd to drive minus-end-directed motility depends critically on the rigidity of the head-proximal part of its coiled-coil tail (often called the 'neck'; the remainder of the tail is called the 'stalk'). Endres *et al*. [8] tested the effects of inserting flexible linkers at the head-tail junction, and within the coiled-coil tail, proving firmly that a two-chain coiled tail is a prerequisite for coupling microtubule-activated ATP turnover to microtubule sliding motility. These results explain why wild-type kinesin-14 likes to dimerize, either with itself, as for Ncd, or, as in Kar3, with a non-motor partner protein (Cik1 or Vik1). Dimerization can stiffen the lever, via coiled-coil formation. The experiments of Endres *et al. *[8] also confirm that the speed of kinesin-14-driven microtubule sliding increases in direct proportion to the predicted lever length.

## Coupling between docking of the kinesin carboxyl terminus and force generation

For the plus-end-directed kinesin-1, kinesin-3 and kinesin-5, crystallography has revealed that the flexible carboxyl terminus of the head (called the 'neck-linker') can dock into a slot on the main part of the head, with the amino terminus lying alongside or wrapping over it. Recent simulations suggest that in kinesin-1, this interaction between the amino and carboxyl termini contributes substantially to the free energy of neck-linker docking [9], lending new life to the proposal, originally made a decade ago [10], that neck-linker docking is itself the force-generating event for kinesin-1, equivalent to the lever arm action proposed for myosins. The carboxyl terminus of kinesin-14 is not seen in existing crystal structures. But in the new kinesin-14 crystal structure of Heuston *et al*. [1], the carboxyl terminus of one of the two heads is seen to anneal into a site on the main part of the head in much the same way as the carboxy-terminal neck-linker of plus-end-directed kinesins. The docking occurs in the head with the 'post-stroke' lever position, suggesting that lever motion and docking are coupled. Both heads in the new structure contain ADP, but the occupancy is reduced in the head with the docked carboxyl terminus and post-stroke lever position. Heuston *et al*. speculate that their post-stroke structure may correspond to a no-nucleotide microtubule-bound state of the motor, so that force generation is coupled to ADP release.

**Figure 3:**
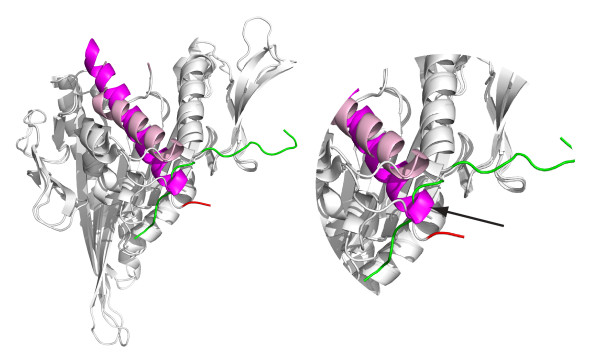
**Docked and undocked positions of the carboxy-terminal neck-linker in kinesin-5**. A kinesin-5 in the docked conformation (PDB 1X88) with a structure in the undocked conformation (PDB 1II6) superimposed. With the alpha4 relay helix in the UP position (shown in pink), the neck-linker (green) can dock into a site on the main part of the motor head. With the alpha4 DOWN (shown in magenta) the neck-linker docking site is sterically blocked, and the neck-linker adopts an alternative position. In the magnified portion of the structure shown on the right, the arrow indicates intrusion of the carboxyl terminus of alpha4 (magenta) into the docking site of the carboxy-terminal neck linker (green).

What is the mechanical significance of the dock-undock cycle of the carboxyl terminus of the kinesin head? How might it be important for both plus- and minus-end-directed motility? Several lines of evidence indicate a coupling between the catalytic status of the active site, the conformation of the alpha4 relay helix, thought to transmit information between the active site and the microtubule-binding interface, and the docking of the carboxy-terminal 'neck-linker' of kinesins in general. Figure 3 shows a superposition of the UP and DOWN states of the alpha4 relay helix in a kinesin-5 crystal structure. With the relay helix in a DOWN state, docking of the carboxy-terminal neck-linker is blocked because the alpha4 helix intrudes into the neck-linker docking site [10]. In the recent literature, the UP state is referred to as 'ATP like' and the DOWN state as 'ADP like', but in my view it remains arguable which chemical state of the active site corresponds to the UP and DOWN states of the alpha4, once the kinesin head is bound to the microtubule. The vast majority of published crystal structures of kinesins have ADP in the motor active site, but both UP and DOWN states of the alpha4 are seen, and there is no correlation, taking the dataset as a whole, between the status of the active site and the conformation of the alpha4 relay helix [11].

Here again, high-resolution cryoelectron microscopy can help. Hirose and colleagues [12] examined Kar3, a minus-end-directed kinesin-14, and found large-scale melting of the alpha4 relay helix on going from the state with bound ATP analog AMPPNP to the empty state, but any effects on the carboxyl terminus or coiled-coil tail are unknown as they were not included in the motor construct used. Movement of the alpha4 helix and docking of the neck-linker has been confirmed in several plus-end-directed kinesins in complex with microtubules [13].

## Rooting out the reversal mechanism

The new work of Heuston and colleagues [1] shows that in kinesin-14, as in other members of its extended family, docking and undocking of the carboxy-terminal residues of the head can occur during ATP turnover. The outstanding problem now is to work out the molecular mechanism by which the coiled-coil lever of kinesin-14 is driven from pre-stroke to post-stroke positions and back again. One possibility is that residues on the microtubule contribute. Whatever the answer may be, it is a safe bet that the kinesin-14 mechanism holds yet more surprises in store.

## References

[B1] Heuston E Bronner CE Kull FJ Endow SA A kinesin motor in a force- producing conformation BMC Struct Biol 2010 10 19 10.1186/1472-6807-10-19 20602775 PMC2906495

[B2] Walker RA Salmon ED Endow SA The *Drosophila *claret segregation protein is a minus-end directed motor molecule Nature 1990 347 780 782 10.1038/347780a0 2146510

[B3] McDonald HB Stewart RJ Goldstein LS The kinesin-like ncd protein of *Drosophila *is a minus end-directed microtubule motor Cell 1990 63 1159 1165 10.1016/0092-8674(90)90412-8 2261638

[B4] Endow SA Waligora KW Determinants of kinesin motor polarity Science 1998 281 1200 1202 10.1126/science.281.5380.1200 9712586

[B5] Endow SA Higuchi H A mutant of the motor protein kinesin that moves in both directions on microtubules Nature 2000 406 913 916 10.1038/35022617 10972296

[B6] Yun M Bronner CE Park CG Cha SS Park HW Endow SA Rotation of the stalk/neck and one head in a new crystal structure of the kinesin motor protein, Ncd EMBO J 2003 22 5382 5389 10.1093/emboj/cdg531 14532111 PMC213785

[B7] Makino T Morii H Shimizu T Arisaka F Kato Y Nagata K Tanokura M Reversible and irreversible coiled coils in the stalk domain of ncd motor protein Biochemistry 2007 46 9523 9532 10.1021/bi700291a 17655278

[B8] Endres NF Yoshioka C Milligan RA Vale RD A lever-arm rotation drives motility of the minus-end-directed kinesin Ncd Nature 2006 439 875 878 10.1038/nature04320 16382238 PMC2851630

[B9] Khalil AS Appleyard DC Labno AK Georges A Karplus M Belcher AM Hwang W Lang MJ Kinesin's cover-neck bundle folds forward to generate force Proc Natl Acad Sci USA 2008 105 19247 19252 10.1073/pnas.0805147105 19047639 PMC2592363

[B10] Vale RD Milligan RA The way things move: looking under the hood of molecular motor proteins Science 2000 288 88 95 10.1126/science.288.5463.88 10753125

[B11] Grant BJ McCammon JA Caves LS Cross RA Multivariate analysis of conserved sequence-structure relationships in kinesins: coupling of the active site and a tubulin-binding sub-domain J Mol Biol 2007 368 1231 1248 10.1016/j.jmb.2007.02.049 17399740

[B12] Hirose K Akimaru E Akiba T Endow SA Amos LA Large conformational changes in a kinesin motor catalyzed by interaction with microtubules Mol Cell 2006 23 913 923 10.1016/j.molcel.2006.07.020 16973442 PMC1635653

[B13] Lang MJ Hwang W Motor proteins: kinesin drives with an underhead cam Curr Biol 2010 20 R408 R410 10.1016/j.cub.2010.03.015 20462483

